# Supplementary material for: An allosteric interaction controls the activation mechanism of SHP2 tyrosine phosphatase
Source: Sci Rep. 2020 Oct 28;10:18530. doi: 10.1038/s41598-020-75409-7 (PMC7595171; doi:10.1038/s41598-020-75409-7)
Supplement: Supplementary file 2 — Supplementary Information 2. [file 41598_2020_75409_MOESM2_ESM.pdf]

Supplementary Information for

An allosteric interaction controls the activation mechanism of SHP2 tyrosine phosphatase

Massimiliano Anselmi[1,2*], Jochen S. Hub[2]

[1]Institute for Microbiology and Genetics, Georg-August-Universität Göttingen, 37077 Göttingen, Germany

[2]Theoretical Physics and Center for Biophysics, Saarland University, Campus E2.6, 66123 Saarbrücken, Germany

[*]corresponding author: manselm@gwdg.de

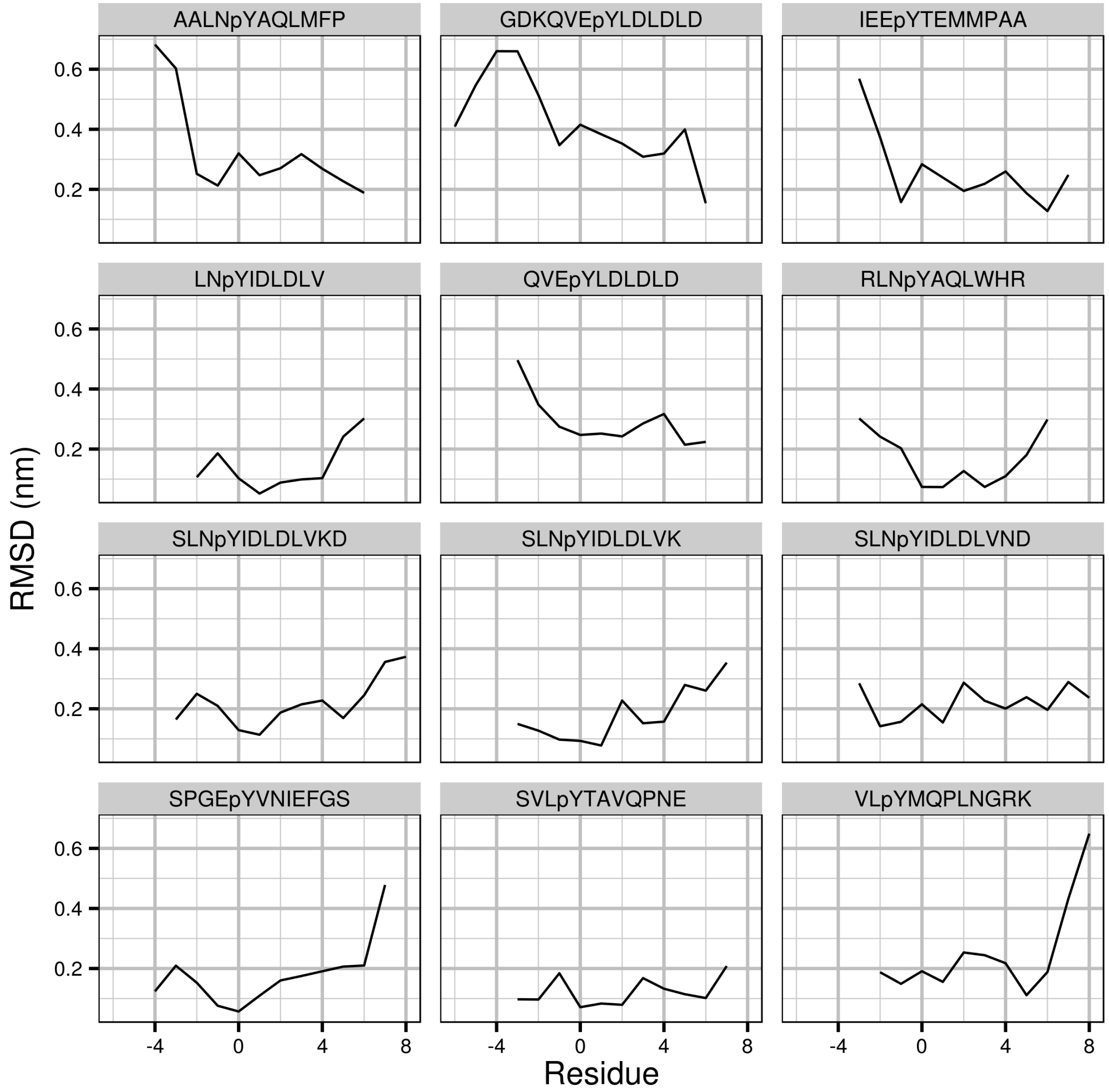

Figure S1. Root mean-square deviation (RMSD) of the most representative MD conformation defined as the central structure of the main structural cluster. The RMSD of the phosphopeptide C_α atoms is given relative the corresponding reference crystal structure, after superimposing only the backbone atoms of the N-SH2 domain.

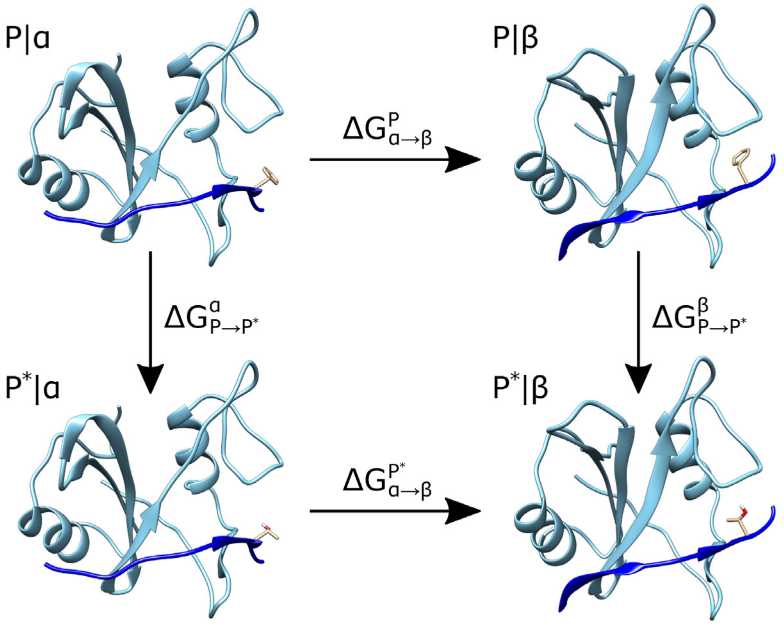

Figure S2. Thermodynamic cycle used for computing the change of preference of the ligand/N-SH2 complex for the α or the β state upon introducing a mutation in the ligand. Change of the stability of the ligand/N-SH2 complex upon introducing a mutation in the ligand, given by the respective free energy difference $\Delta G_{P\rightarrow P*}$, where P and $P*$ denote the initial and the alchemically transformed amino acid, respectively. To quantify the effect of the $P \rightarrow P^*$ mutation on the N-SH2 preference for the α versus the β state, we computed $\Delta G_{P\rightarrow P*}$, twice for each mutation, namely with N-SH2 restrained either in the α or in the β state, denoted $\Delta G^{\alpha}_{P\rightarrow P*}$ and $\Delta G^{\beta}_{P\rightarrow P*}$, respectively. Hence, the difference $\Delta\Delta G = \Delta G^{\beta}_{P\rightarrow P*} - \Delta G^{\alpha}_{P\rightarrow P*}$ indicates the change of preference of the ligand/N-SH2 complex for the α or the β state, where a positive $\Delta\Delta G$ indicates an augmented preference for the α state, and a negative $\Delta\Delta G$ an augmented preference for the β state. Critically, the $\Delta\Delta G$ values represent purely the change of the α-versus-β population for a bound ligand.

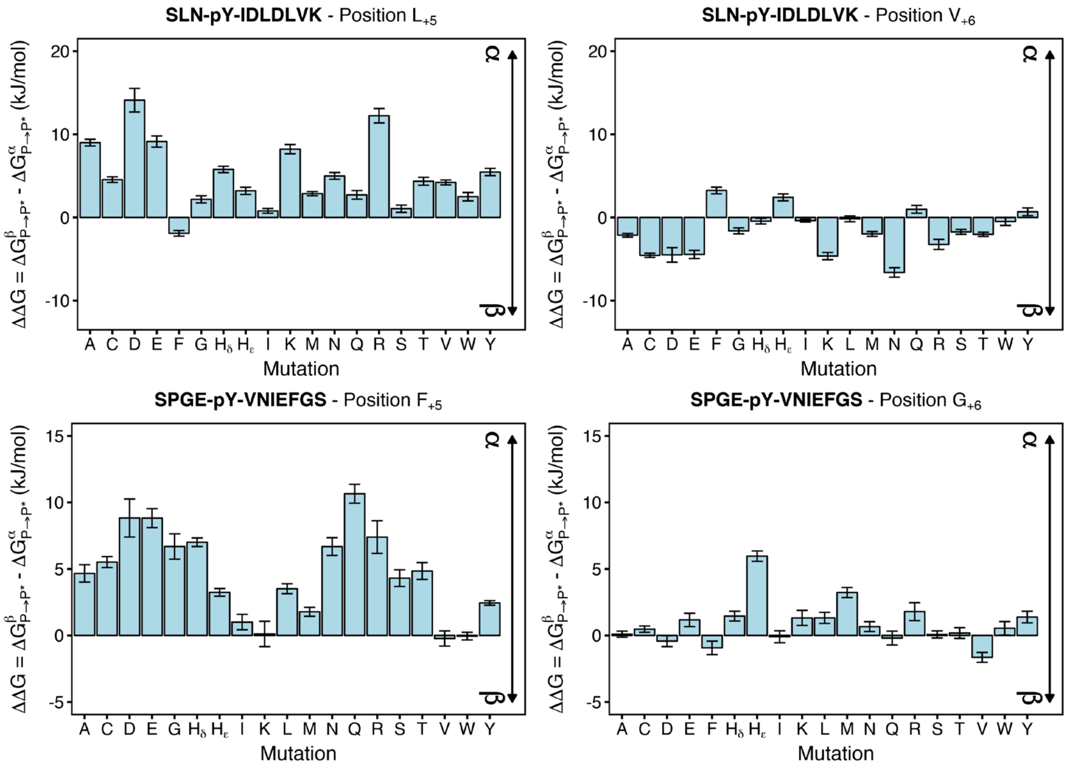

Figure S3. Change of preference of the ligand/N-SH2 complex for the α or the β state upon introducing a mutation in the ligand. Positive values of $\Delta\Delta G$ indicates an augmented preference for the α state respect to the reference sequence, and a negative $\Delta\Delta G$ an augmented preference for the β state. The calculations were performed on the position +5 and +6 of two ligands, SLNpYIDLDLVK and SPGEpYVNIEFGS, yielding the analogs SLNpYIDLDX_{+5}VK, SLNpYIDLDLX_{+6}K, SPGEpYVNIEX_{+5}GS and SPGEpYVNIEFX_{+6}S, where X considers all possible substitutions with the exception of proline.

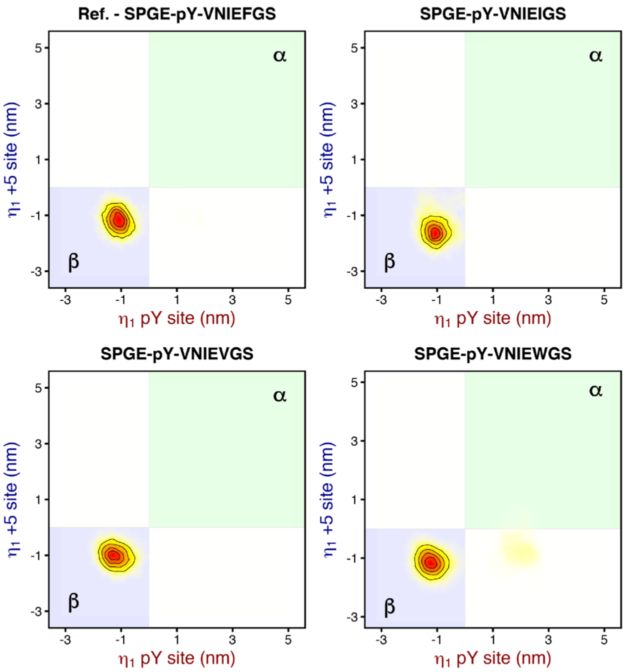

Figure S4. Projection distributions of the trajectory of the N-SH2 domain bound to the reference SPGEpYVNIEFGS peptide and its mutants at position +5, SPGEpYVNIEX$_{+5}$GS (X = I, V, W), on the principal component plane defined by the principal component eigenvector describing the motion of the pY loop and of the +5 site. The region of the plane corresponding to the α state (pY loop closed, +5 site closed) is shaded in green, whereas that of the β state (pY loop open, +5 site open) is shaded in blue. The substitution of the phenylalanine residue at position +5 in SPGEpYVNIEFGS respectively in isoleucine (I), valine (V), and tryptophan (W) retains the peptide binding mode to select the β state.

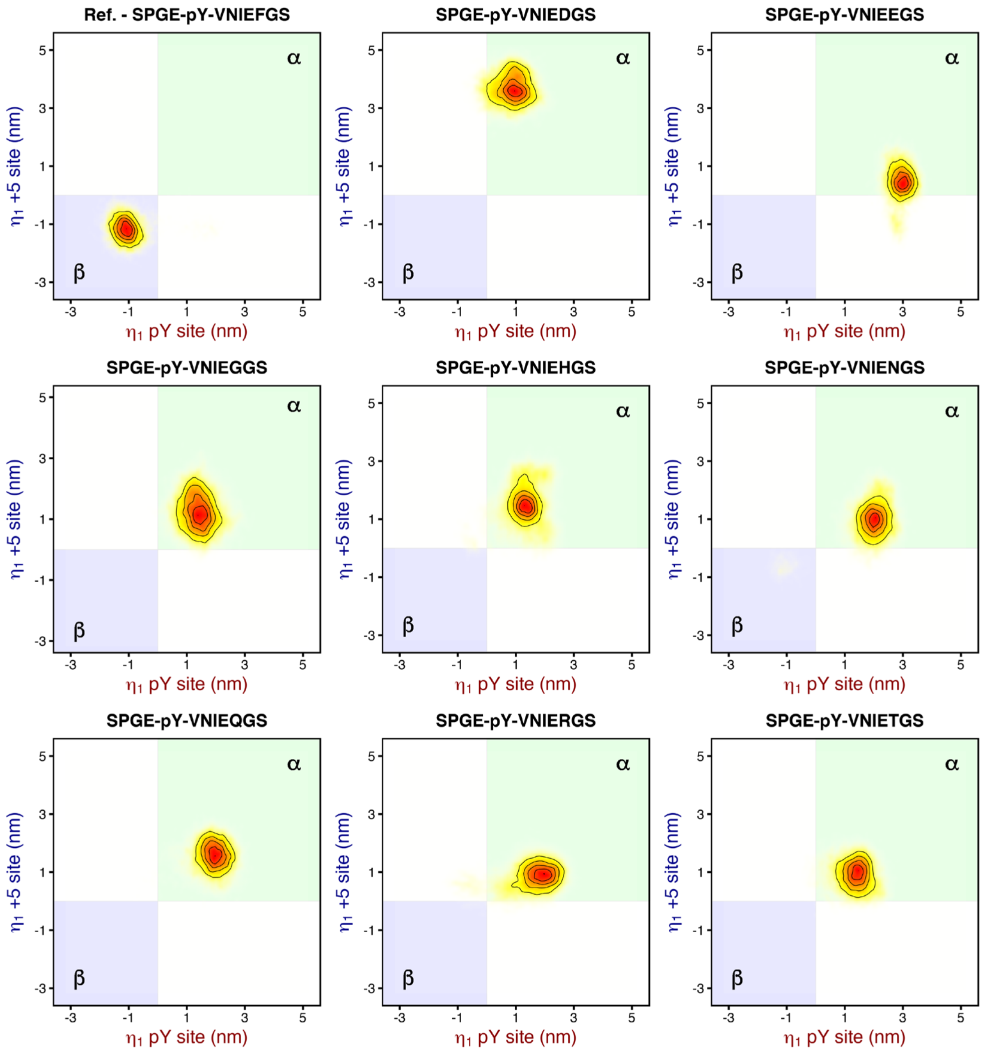

Figure S5. Projection distributions of the trajectory of the N-SH2 domain bound to the reference SPGEpYVNIEFGS peptide and its mutants at position +5, SPGEpYVNIEX$_{+5}$GS (X = D, E, G, H, N, Q, R, T), on the principal component plane defined by the principal component eigenvector describing the motion of the pY loop and of the +5 site. The region of the plane corresponding to the α state (pY loop closed, +5 site closed) is shaded in green, whereas that of the β state (pY loop open, +5 site open) is shaded in blue. The substitution of the phenylalanine residue at position +5 in SPGEpYVNIEFGS respectively in aspartate (D), glutamate (E), glycine (G), histidine (H$_{\delta}$), asparagine (N), glutamine (Q), arginine (R), and threonine (T) turns the peptide binding mode from selecting the β state to favoring the α state.

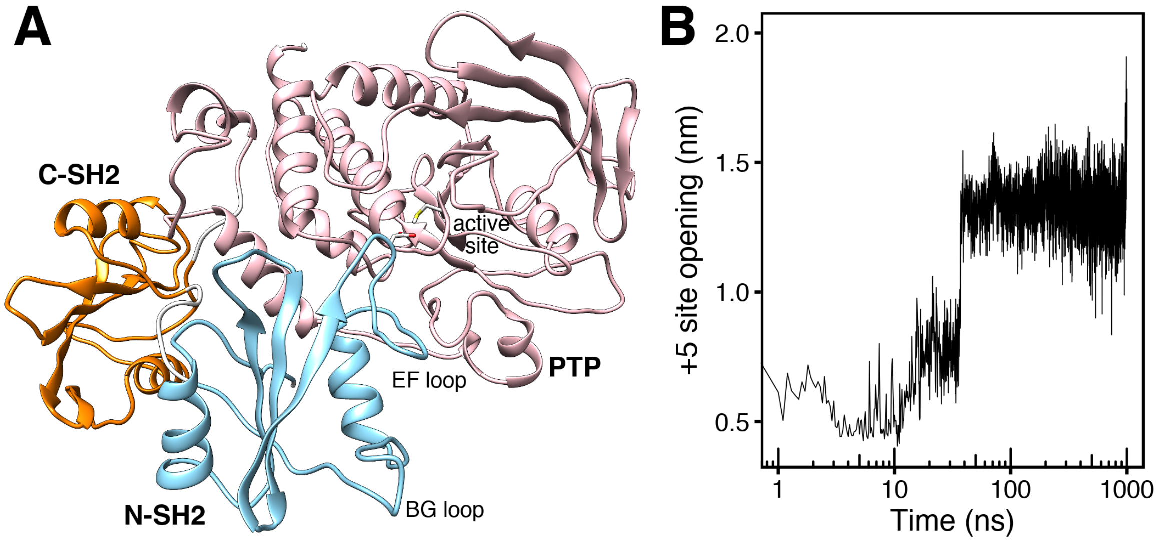

Figure S6. (A) Representative structure of autoinhibited SHP2 equilibrated in solution. The pY loop and the +5 site, delimited by the EF and BG loop, adopt an open conformation, consistent with the β state. Residues Asp^{61} (belonging to the N-SH2 blocking loop) and Cys^{459} (belonging to the catalytic PTP loop) are depicted in sticks (B) Trajectory of the +5 site opening (Gly^{67} C_{α}– Lys^{89} C_{α} distance) during the equilibration run (1 μs).

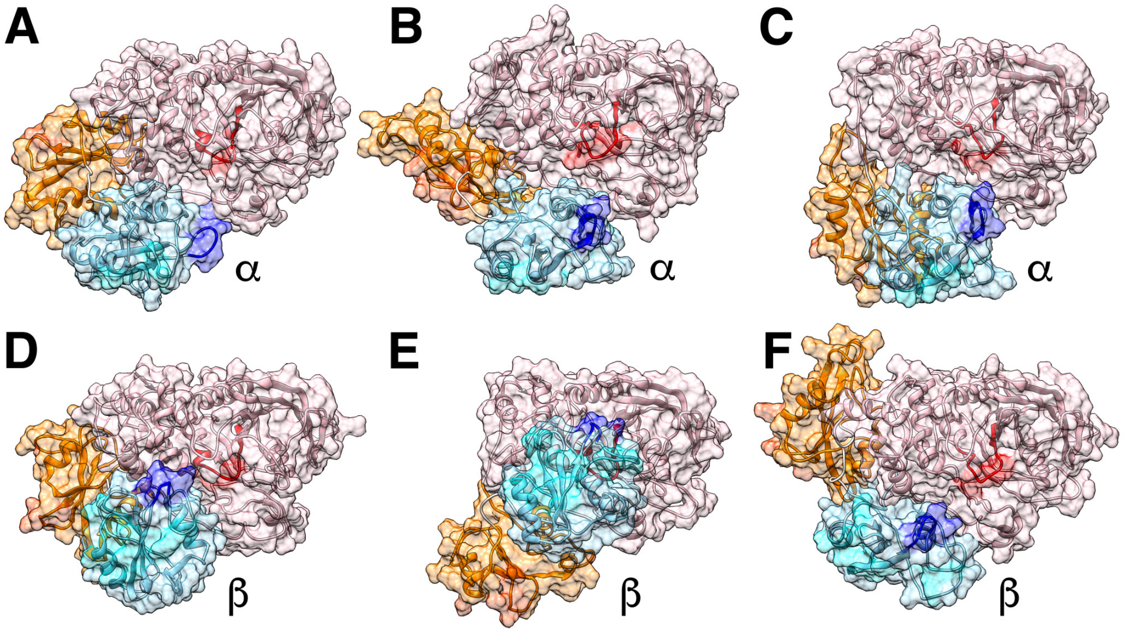

Figure S7. Open and active structures obtained by pulling simulations coupled with simulated tempering, restraining the N-SH2 domain (cyan cartoon) either to the α (A-C) or to the β state (D-F). The final position of N-SH2 differs among independent simulation runs, indicative of a large accessible conformational space of activated SHP2. The N-SH2 domain is colored in cyan, the C-SH2 domain in orange, PTP domain in pink, the PTP catalytic site in red, and the N-SH2 blocking loop in blue.

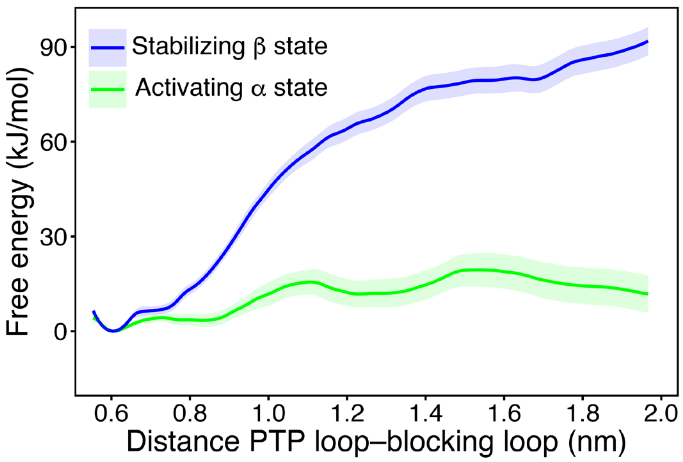

Figure S8. Free energy profiles for the opening of SHP2 using an alternative reaction coordinate (RC_2), with the N-SH2 domain restrained in the activating α state (green line) or in the stabilizing β state (blue line). RC_2 was defined as the distance between the C_α atoms of residues Asp^{61} and Ala^{461}. The profiles are qualitatively similar to the profiles computed with RC_1.

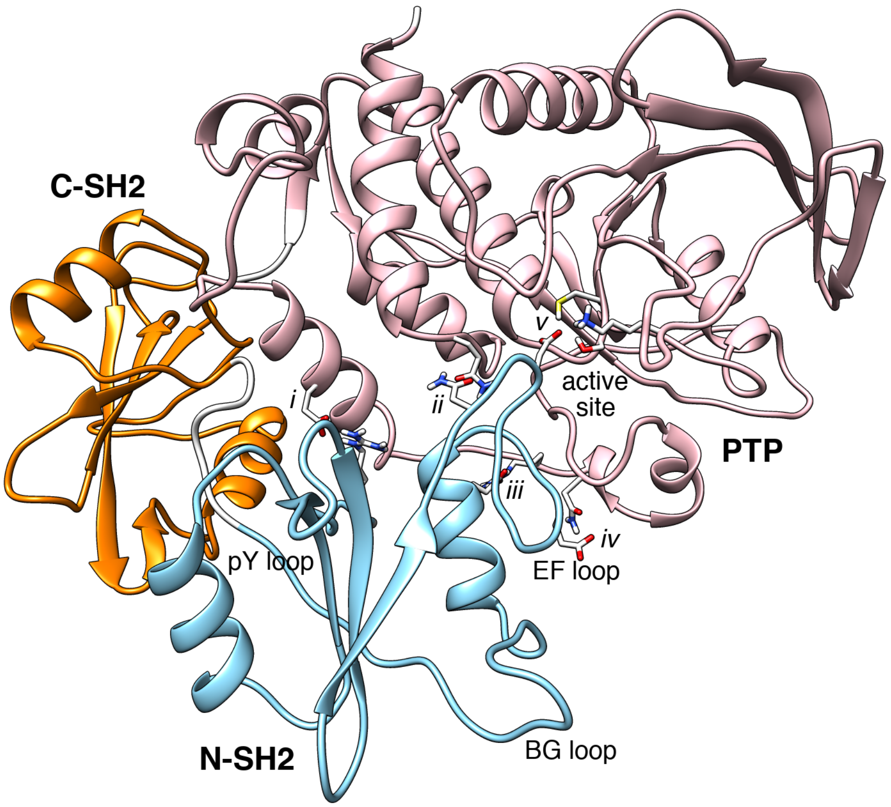

Figure S9. Representative structure of autoinhibited SHP2 with the N-SH2 domain restrained in the α-state. The equilibration procedure allowed to mostly preserve the interactions between the N-SH2 blocking loop and the catalytic PTP loop, as well as the salt bridges and H-bonds between the following pairs of residues: Arg^{4}–Glu^{252} *(i)*, Asn^{58}–Gln^{506} *(ii)*, Glu^{76}–Arg^{265} *(iii)*, Glu^{69}–Asn^{281} *(iv)*, Asp^{61}–Ser^{460} and Asp^{61}–Lys^{366} *(v)*, as labelled in the figure. Hence, restraining N-SH2 to the α state leads only to a moderate weakening of the N-SH2–PTP interactions but not to immediate N-SH2 dissociation.

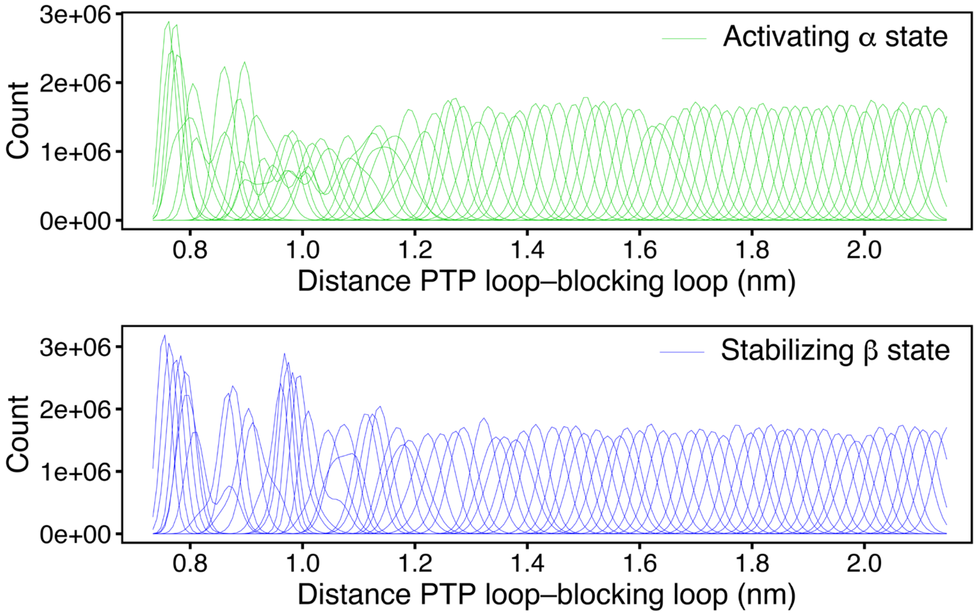

Figure S10. Umbrella histograms used for calculating the free energy profiles along the reaction coordinate RC_1, with the N-SH2 domain restrained in the activating α state (green lines) or in the stabilizing β state (blue lines). Evidently, the histograms well overlap, as required for reliable free energy profile calculations.

Table S1. Degree of openness of the central beta strands βC and βD in various crystal structures, quantified by the distance between the backbone carbonyl oxygen of Gly^{39} and the backbone nitrogen atom of Asn^{58}. The structures of isolated N-SH2 bound to peptides are shaded in green. The structures of wild type SHP2 and mutants are shaded in light and dark blue respectively. Other structures are shaded in yellow.

Pdb ID	Distance $GLY^{39}O$-$ASN^{58}N$ (Å)	Description	Notes
1AYA	7.225	N-SH2 only / bound to high affinity peptide	
1AYB	6.611	N-SH2 only / bound to high affinity peptide	
1AYC	7.558	N-SH2 only / bound to low affinity peptide	
3TL0	7.679	N-SH2 only / bound to low affinity peptide	
4QSY	7.356	N-SH2 only / bound to low affinity peptide	
2SHP	3.722	SHP2	
4DGP	3.451	SHP2	
4DGX	3.499	SHP2 Y279C mutant	
4GWF	4.020	SHP2 Y279C mutant	BG loop incomplete
4H1O	4.749	SHP2 D61G mutant	BG loop incomplete
4H34	4.803	SHP2 Q506P mutant	BG loop incomplete
4NWF	3.098	SHP2 N308D mutant	BG loop incomplete
4NWG	4.877	SHP2 E139D mutant	BG loop incomplete
4OHD	2.956	SHP2 A461T mutant	
4OHE	4.322	SHP2 G464A mutant	
4OHH	4.112	SHP2 Q506P mutant	
4OHI	4.333	SHP2 Q510E mutant	
4OHL	4.116	SHP2 T468M mutant	
5I6V	3.042	SHP2 F285S mutant	BG loop incomplete
5IBM	3.477	SHP2 S502P mutant	BG loop incomplete
5IBS	3.755	SHP2 E76Q mutant	BG loop incomplete
1AYD	5.244	N-SH2 only / unbound	
4JE4	5.207	Crystal Structure of Monobody NSa1/SHP2 N-SH2 Domain Complex	
5DF6	4.947	tandem SH2 domains in complex with a TXNIP peptide	
5EHP	4.429	SHP2 in Complex with Allosteric Inhibitor SHP836	PO4 bound
5EHR	4.455	SHP2 in Complex with Allosteric Inhibitor SHP099	PO4 bound

Table S2. Modeling of initial conformations from PDB structures

Initial structures of N-SH2/ligand complexes were generated from crystal structures with PDB codes listed in the table. To this end, PDB structures with similar ligand sequence were modified by means of Molecular Operative Environment (MOE), followed by a local energy minimization with side chain repacking and visual analysis. The substituted residues are highlighted in red.

Simulated Target Sequence	Original Sequence	PDB Code	Target Sequence Source
SLNpYIDLDLVK	GDKQVEpYLDLDLD	4qsy	IRS-1 (rat) 1169-1179
LNpYIDLDLV	GDKQVEpYLDLDLD	4qsy	IRS-1 (rat) 1170-1178
AALNpYAQLMFP	SVLpYTAVQPNE	1aya	Synthetic (ref. 42)
SPGEpYVNIEFGS	SPGEpYVNIEFGS	1ayb	IRS-1 (human) 892-903
VLpYMQPLNGRK	SVLpYTAVQPNE	1aya	Synthetic (ref. 43)
IEEpYTEMMPAA	SVLpYTAVQPNE	1aya	IRS-1 (human) 548-557
SVLpYTAVQPNE	SVLpYTAVQPNE	1aya	PDGFRB (human) 1006-1016
RLNpYAQLWHR	RLNpYAQLWHR	3tl0	Synthetic (ref. 42)
GDKQVEpYLDLDLD	GDKQVEpYLDLDLD	4qsy	GAB1 (human) 621-633
QVEpYLDLDLD	GDKQVEpYLDLDLD	4qsy	GAB1 (human) 624-633
